# Serotonin Syndrome With Concomitant Antidepressant and Tramadol: A Case Report

**DOI:** 10.7759/cureus.76968

**Published:** 2025-01-05

**Authors:** Pedro Ferreira de Lemos, Rute Fidalgo Maia

**Affiliations:** 1 Unidade de Saúde Familiar (USF) Prelada, Unidade Local de Saúde de Santo António, Porto, PRT

**Keywords:** serotonin-norepinephrine reuptake inhibitor (snri), serotonin syndrome (ss), serotonin toxicity, tramadol, venlafaxine

## Abstract

Serotonin syndrome (SS) is a potentially life-threatening condition that results from the excess of serotonergic activity in the central nervous system. It is classically described as a triad of neuromuscular abnormalities, altered mental status, and autonomic hyperactivity; these are, most often, due to iatrogenic drug interactions or intentional overdose.

This case study is about a 58-year-old woman who was being treated with venlafaxine and tramadol due to her past medical history of major depressive disorder and neuropathic pain. These symptoms appeared after a mastectomy, which was performed to treat an invasive ductal carcinoma. The sudden increase of the antidepressant medication and the interaction with another drug that inhibits serotonin reuptake (tramadol) led to tremor, hyperreflexia, diarrhea, tachycardia, hypertension, and fever, meeting Hunter’s criteria for diagnosis of serotonergic syndrome. She received supportive care and diazepam, and the offending agents were removed. The signs and symptoms were reverted in less than 24 hours.

An increased use of agents affecting the serotonergic system to treat different medical conditions raises the likelihood of a higher prevalence and clinical impact of SS in the future. For this reason, it is crucial to raise awareness of this condition to prevent adverse outcomes.

## Introduction

Serotonin syndrome (SS) is a potentially life-threatening iatrogenic condition that results from the excess of serotonergic activity in the central nervous system. It can occur in cases of intentional self-poisoning, interactions between drugs, or, less commonly, due to the action of a single drug [1]. Oates and Sjoerdsma, in 1960, described SS for the first time in patients receiving a monoamine oxidase inhibitor (MAOI) [2].

This condition’s clinical spectrum ranges from mild to lethal, and the classic triad of features includes neuromuscular abnormalities, altered mental status, and autonomic hyperactivity [3], most often presented within 24 hours of medication initiation, overdose or change of dose in a serotoninergic drug [4].

The diagnosis of SS depends on clinical findings, but there are several criteria for this diagnosis, with Hunter’s criteria being the most frequently used [3]. A study found that 85% of general practitioners were unaware of SS [5], so this case report aims to raise awareness of this condition to prevent adverse outcomes.

## Case presentation

This case is about a 58-year-old female with a past medical history of major depressive disorder, invasive ductal carcinoma, and neuropathic pain after a partial mastectomy. As regular medications, she has been taking venlafaxine 75 mg, zolpidem 10 mg, letrozole 2.5 mg, pregabalin 50 mg (twice daily), and tramadol 50 mg (twice daily).

The patient attended a psychiatrist appointment in her chronic pain unit due to her nearly daily low mood, fatigue, loss of engagement in any activity of the day, insomnia, poor concentration, feeling worthless, and recurrent suicidal ideation without a specific plan or attempt. She correlated these feelings with her divorce and the fact that she did not currently have any family support (her only daughter emigrated). For this reason, the psychiatrist decided to increase the venlafaxine dose from 75 to 150 mg once per day.

The next day, she asked for an urgent appointment with her family doctor because of a one-day history of tremor, clonus, diaphoresis, and diarrhea. During the physical exam, she was agitated, with generalized tremors, hyperreflexia, inducible clonus, tachycardia (115 bpm), hypertension (158/94 mmHg), and fever (38°C). The practitioner suspected serotonergic syndrome, and the patient was referred to an emergency service. The clinical presentation was subsequently discussed with her psychiatrist, who decided to remove venlafaxine and tramadol and prescribe her 10 mg of diazepam. The patient refused to be hospitalized, so an appointment was made for her the following day, and symptoms had resolved by then.

## Discussion

SS is a rare condition that results from the excess of serotonergic activity in the central nervous system, usually due to serotonergic polypharmacy and after initiation or dose escalation of one of the agents. It has been widely demonstrated to be caused with antidepressants, such as MAOIs, selective serotonin reuptake inhibitors (SSRIs), and serotonin and norepinephrine reuptake inhibitors (SNRIs), but it can occur with any other drug classes that increase serotonin toxicity, such as analgesics.

Tramadol is a centrally acting opioid analgesic that has a dual mechanism of action. Even though it primarily acts as an agonist of the mu-opioid receptor, it also inhibits serotonin and norepinephrine reuptake, enhancing the analgesic effect. Therefore, this drug has a recognized risk of causing a serotonergic syndrome [6].

In this case, the concomitant administration of two different serotonergic drugs while simultaneously increasing the dose of one agent led to the development of the classic triad of the serotonergic syndrome: mental status change (agitation), neuromuscular abnormalities (tremor, hyperreflexia, and clonus), and autonomic hyperactivity (tachycardia, hypertension, fever, diaphoresis, and diarrhea), within 24 hours (Table 1).

**Table 1:** Symptoms/signs of the patient within the classic triad

Mental status change	Autonomic hyperactivity	Neuromuscular abnormalities
Agitation	Tachycardia, hypertension, fever, diaphoresis, and diarrhea	Tremor, hyperreflexia, and clonus

After reviewing the patient’s regular medications (excluding antipsychotics, substance abuse, and withdrawal) and conducting a thorough physical examination, it was possible to rule out various diagnoses, including neuroleptic malignant syndrome, malignant hyperthermia, anticholinergic toxicity, and other forms of intoxication [3].

This patient presentation satisfied the most sensitive and specific criteria for diagnosing this syndrome, Hunter’s toxicity criteria decision rules (Table 2), via the clinical findings of inducible clonus, agitation, diaphoresis, tremor, hyperreflexia, and a temperature of >38°C [7].

**Table 2:** Hunter’s toxicity criteria decision rules Reference [7].

Hunter Serotonin Toxicity Criteria: decision rules
In the presence of a serotonergic agent
1. IF (spontaneous clonus = yes) THEN serotonin toxicity = YES
2. ELSE IF (inducible clonus = yes) AND [(agitation = yes) OR (diaphoresis = yes)] THEN serotonin toxicity = YES
3. ELSE IF (ocular clonus = yes) AND [(agitation = yes) OR (diaphoresis = yes)] THEN serotonin toxicity = YES
4. ELSE IF (tremor = yes) AND (hyperreflexia = yes) THEN serotonin toxicity = YES
5. ELSE IF (hypertonic = yes) AND (temperature > 38C) AND [(ocular clonus = yes) OR (inducible clonus = yes)] then serotonin toxicity = YES
6. ELSE serotonin toxicity = NO

Management of mild cases, such as this one, consists of removing all serotonergic agents, introducing supportive care, and using benzodiazepines. Symptoms and signs usually resolve within 24 hours after removal of the causing agents; this clinical case followed this principle. Treatment with a serotonin antagonist (cyproheptadine) could be required in moderately ill patients [3].

With the rise in life expectancy and the consequent higher prevalence of chronic diseases, polypharmacy is becoming increasingly common, potentially resulting in harmful drug interactions such as the aforedescribed.

## Conclusions

Despite SS being a rare condition, its potentially life-threatening nature requires prompt recognition and investigation by physicians.

The present case report is consistent with a diagnosis of mild SS, successfully managed through the withdrawal of the causative agents, venlafaxine and tramadol, and the administration of a benzodiazepine.

Serotonergic drugs are used in psychiatric disorders but can also be prescribed in multiple other medical diagnoses, including the management of chronic pain. Consequently, the concomitant use of tramadol with antidepressants is highly prevalent, making it essential to raise awareness of SS among all physicians to prevent adverse outcomes.

## Figures and Tables

**Figure 1:**
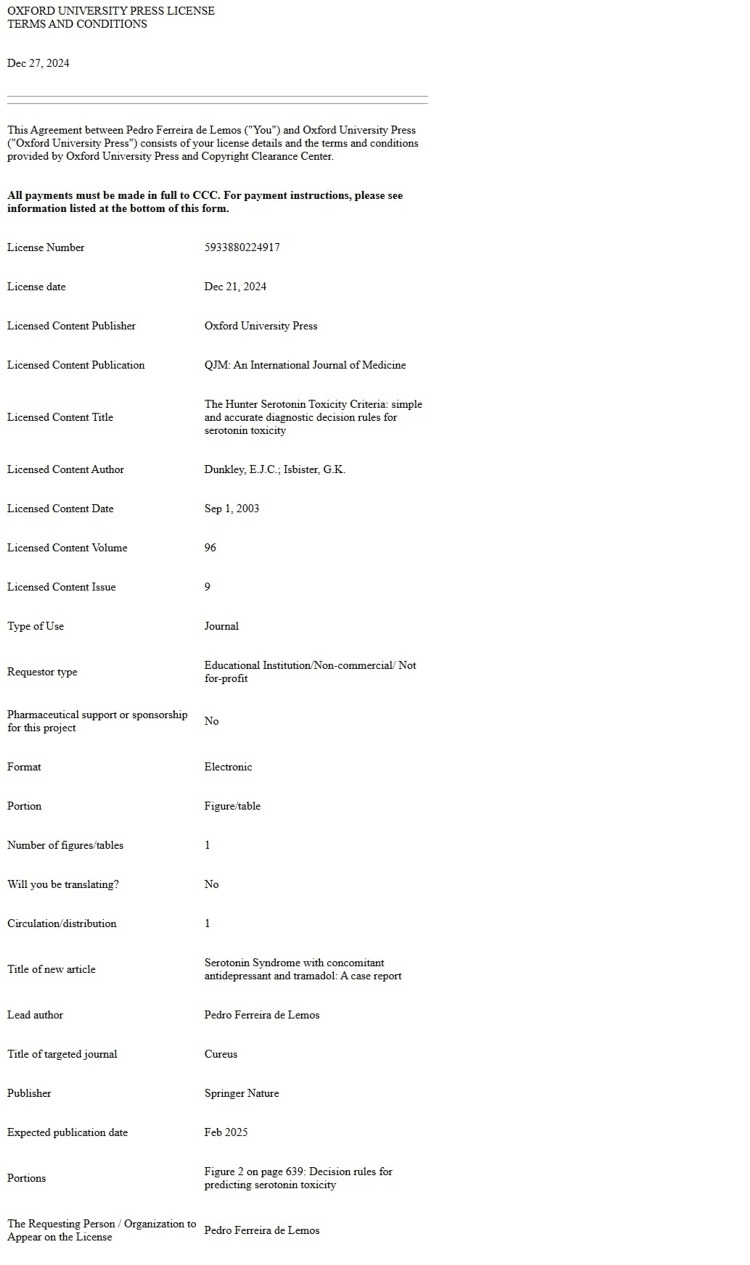
Permission document for Table 2
